# Cervical arthroplasty with ROTAIO® cervical disc prosthesis: first clinical and radiographic outcome analysis in a multicenter prospective trial

**DOI:** 10.1186/s12891-016-0880-7

**Published:** 2016-01-12

**Authors:** J. Obernauer, J. Landscheidt, S. Hartmann, G. A. Schubert, C. Thomé, C. Lumenta

**Affiliations:** Department of Neurosurgery, Innsbruck Medical University, Anichstrasse 35, 6020 Innsbruck, Austria; Klinik f. Neurochirurgie, Klinikum Bogenhausen, Munich, Germany; Department of Neurosurgery, RWTH Aachen University, Aachen, Germany

**Keywords:** Cervical disc replacement, Cervical disc arthroplasty, Range of motion, Spinal instrumentation

## Abstract

**Background:**

Cervical Disc Arthroplasty (CDA) seems to be an alternative to Anterior Cervical Decompression and Fusion (ACDF) and was developed to minimize the risk of Adjacent Segment Disease (ASD). The ROTAIO Cervical Disc Prosthesis represents a new unconstrained implant with a variable centre of rotation which should enable physiological facet-guided movement. The aim of this current study was to evaluate the clinical outcomes after arthroplasty using ROTAIO Cervical Disc Prosthesis.

**Method:**

Twenty-seven female and 18 male patients (*n* = 45) with a mean age of 43.7 ± 7.8 years were prospectively followed up for a maximum of 24 month. Clinical outcomes were assessed by Neck Disability Index (NDI), visual analogue scale (VAS) scores for neck and arm pain, patients´ overall satisfaction and the usage of analgesics. Additionally, radiographic information including ROM of the functional spinal unit (FSU) and signs of adjacent segment disease were recorded.

**Results:**

NDI and VAS scores showed significant improvement 6 months after surgery and at last follow-up (*p* < 0.001). Concerning overall satisfaction 95.7 % of the patients showed good to excellent results at the last visit and a significant reduction of analgesic usage was observed (*p* < 0.001). Radiographic measurements showed a mean increase of ROM up to 8.40° in the treated FSU at last follow-up (*p* < 0.001). No signs of anterior migration or dislocation of the prosthesis and no subsidence was recorded radiographically. There were no major complications and a low rate of secondary procedures (2.2 %).

**Conclusion:**

In the 24-months follow-up the ROTAIO Cervical Disc Prosthesis provided excellent clinical and radiographical results and seems to be safe and effective for the treatment of symptomatic single-level degenerative disc disease.

## Background

Cervical spondylosis and degenerative disc disease may result in compression of one or more nerve roots and/or the spinal cord. If non-operative treatment fails, anterior cervical discectomy and fusion (ACDF) is a common treatment for cervical radiculopathy and myelopathy [1]. The procedure shows high patient satisfaction scores and a 95 % arthrodesis rate [2, 3]. Nevertheless the technique causes a loss of segmental motion and a possible need for reoperation because of pseudoarthrosis or adjacent segment degeneration (ASD). In recent years, cervical disc replacement (CDR) has become popular as an alternative to ACDF. Its theoretical and observed advantages include a more physiologic distribution of range of motion (ROM), reduced adjacent level stresses and a lowered rate of ASD. Currently, there are many commercially available artificial discs with different biomechanical properties [3–7].

This study reports the first results of a prospective, two-centre observational clinical trial on a newly developed artificial disc replacement device. There are currently no long term reports about the ROTAIO Cervical Disc Prosthesis (SIGNUS Medizintechnik GmbH, Alzenau, Germany). The study objective was to evaluate the clinical and radiological outcome after a maximum follow up of two years and to compare the results to available data of other artificial disc devices.

## Methods

The observation was institutional review board approved (Ethics Commission, Medical University Innsbruck – Austria). All patients at the two centres who met the inclusion/exclusion criteria provided written informed consent and were included in the trial. Patient selection was based on the following inclusion and exclusion criteria. Inclusion criteria: (1) 18 to 65 years of age, (2) a single cervical disc disease between the level C3 and C7, (3) failed conservative treatment of at least 6 weeks or in case of progressive nerve root or spinal cord compression and (4) a minimum Neck Disability Index (NDI) of 15 points (30 %). Exclusion criteria: (1) cervical instability defined by translation on flexion–extension radiographs compared to the adjacent level, (2) non-mobility of the level, (3) fused adjacent levels, (4) neck pain as the solitary symptom, (5) prior surgery at target level, (6) metabolic bone disease or endocrine disorders, (7) pregnancy, (8) traumatic injury of spine, (9) HIV, metastatic cancer, presence of infection and (10) allergy to materials used in the device.

### Demographic data

From January 2011 to December 2012, CDRs using the ROTAIO Cervical Disc Prosthesis were performed in 45 patients. All patients were successfully included, 28 patients had their last follow-up 12 months postoperatively and 17 patients were available for 24 months follow-up (mean 16.5 ± 5.8 month) . There were 27 female and 18 male patients with a mean age of 43.7 ± 7.1 years (range: 28–65 years). Seven patients were treated at C4/5, 27 patients at C5/6 and 11 patients at C6/7. All patients showed radiculopathic disorders, 4 of them in combination with mild signs of myelopathy.

### Device design

**Fig. 1:**
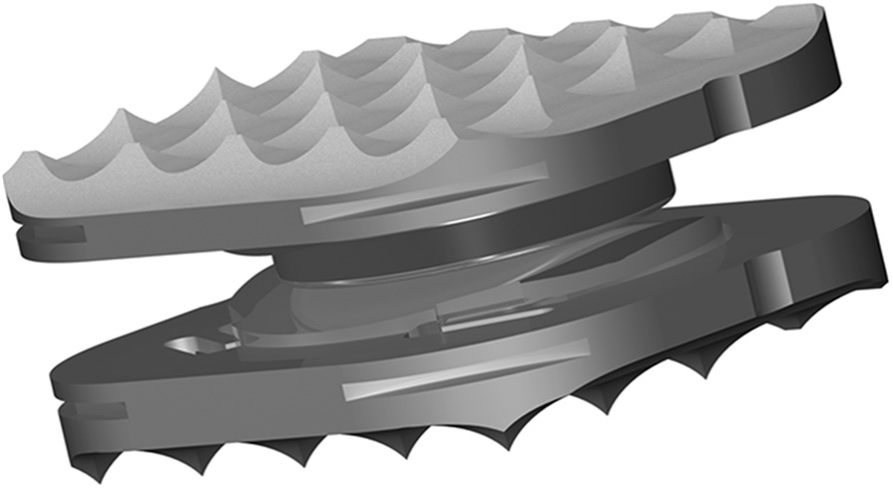
ROTAIO Cervical Disc Prosthesis (Source: SIGNUS Medizintechnik GmbH)

The ROTAIO Cervical Disc Prosthesis (Fig. 1) is intended to be used for disc replacement in the cervical spine (C3-C7). In addition to restoring disc height, its primary function is to maintain physiological movement in the affected segment. The ROTAIO Cervical Disc Prosthesis represents a new unconstrained implant with a variable centre of rotation which should enable physiological facet-guided movement. For maximum coverage of the vertebral body end plates a total of 16 different sizes are available with various surface areas and heights. The prosthesis is pre-mounted on an insertion bracket to avoid the need for intraoperative assembly. Instruments specially developed for use with the implant system are available to ensure safe application.

The prosthesis consists of a superior and an inferior end plate (Titanium alloy to ISO 5832-3) on which the sliding elements (Cobalt-chrome alloy to ISO 5832-12) are anchored and secured by means of a fixation pin. The implant design provides an optimum fit to the anatomy of the intervertebral space. Assembly of ROTAIO is done in an axial direction. This has the effect that the slide plate is radially surrounded by the cover plate, which prevents the sliding plate from dislodgement. For optimum primary stabilization the end plates have a toothed surface. A special blasting treatment of the end plates increases surface area and thus bony integration.

### Surgical technique

A standard right-sided anterior approach was routinely performed. The surgical technique was similar to those for a routine anterior cervical decompression. Implantation of the prosthesis was performed strictly following the manufacturer’s specifications and users´ manual. The endplate preparation was - corresponding to the design of the implant - performed using a high-speed drill followed by curetting the endplates with the manufacture´s trial-implant. The posterior longitudinal ligaments were completely removed in all cases. Any remaining superior posterior osteophytes overhanging the endplate were removed. All cases were treated by neurosurgical specialists who attended manufacture´s instruction lectures.

### Clinical outcome

Clinical evaluations were performed using standardized questionnaires preoperatively, postoperatively before discharge, 3 to 6, 12 and 24 months after implantation. For the clinical outcome validated self-assessment outcome measures including neck disability index (NDI), patient satisfaction index (PSI), visual analogue scale (VAS) for neck and arm pain and patients overall satisfaction were recorded. Additionally the use of non-steroidal anti-inflammatory drugs (NSAID) was assessed. The NDI ranged from 0 to 50 and VAS scores from 0 to 10. Concerning PSI patients had to answer the question, “Would you like to have the same treatment for the same ailment again?” with (1) *definitely yes,* (2) *probably yes,* (3) *probably not* or (4) *definitely not*. The use of NSAIDs was defined as: (1) *never,* (2) *seldom*; 1 to 3 times a week, (3) *often*; 3 to 5 times a week or (4) *always*; 1 or more times a day. The preoperative scores were compared with the follow-up scores. To analyse overall satisfaction, the patient had to rate their improvement in (1) *excellent,* (2) *significantly better,* (3) *good,* (4) *mild,* (5) *no improvement* or (6) *worsening of ailment*. Complications were intended to be recorded during surgery and at final follow-up.

### Radiographic outcome

Radiographic imaging observed the prosthesis position and function, possible signs of fusion or adjacent segment degeneration in neutral lateral and dynamic flexion and extension X-rays. To evaluate angular range of motion within the treated functional unit the Cobb-technique on dynamic lateral radiographs was used. For each measurement the mean of two observers were calculated and recorded for analysis. In four patients (index level C6/7) radiographic assessment was not possible because of prominent shoulders obscuring the images. All measurements were performed on source-digitalized images using IMPAX EE imaging software (AGFA HealthCare N.V., Belgium).

### Statistical analysis

Statistical analyses were performed using IBM SPSS Statistics Version 20. The trial was designed to detect an absolute difference between pre- and postoperative data of one standard deviation with a power of 80 % at a two sided significance level (probability (p)-value) of 0.05 and a maximal dropout rate of 20 %. Comparisons were performed with the use of an unpaired t-test, or in case of nonparametric values with a Mann-Whitney-U test. Because data analysis did not show a statistically significant difference (*p* > 0.05) between the 12 months and the 24 months follow-up the results are presented combined under the term “last follow up”.

## Results and discussion

### Clinical evaluation

#### Neck disability index

At every time point postoperatively the patients were significantly improved from their preoperative NDI-scores (Table 1). The mean NDI had improved from 21.6 ± 9.8 preoperatively to 10.6 ± 8.3 at 3 to 6 months postoperative (*p* < 0.001) and to 10.5 ± 9.3 (*p* < 0.001) at last follow-up, with an average improvement rate of 51.5 %. A significant reduction of NDI scores was reached within 4 to 6 weeks after treatment and reached a plateau by 3 to 6 month after surgery.

**Table 1:** Clinical Results

	Preoperative	3 to 6 month	Last follow-up
NDI (range 0-50)	21.65 ± 9.84	10.65 ± 8.32 (*p* < 0.001)	10.50 ± 9.35 (*p* < 0.001)
VAS neck pain (range 0-10)	5.30 ± 2.81	2.49 ± 1.86 (*p* < 0.001)	3.33 ± 2.75 (*p* < 0.001)
VAS arm pain (range 0-10)	5.93 ± 3.174	1.91 ± 1.81 (*p* < 0.001)	2.88 ± 2.72 (*p* < 0.001)
NSAID –use (% of patients)			
Never	18.5 %	48.9 %	45.8 %
Seldom	14.8 %	23.3 %	33.3 %
Often	14.8 %	16.3 %	8.3 %
Always	51.9 %	9.3 %	12.5 %
		(*p* < 0.001)	(*p* < 0.001)

NOTE. NDI and VAS are provided as mean ± standard. NSAID-use is provided as percentage of patients

#### Neck pain

Significant improvement of the neck pain score occurred early postoperatively to 6 months postoperatively and was maintained at last follow-up (Table 1). The mean preoperative score for neck pain was 5.3 ± 2.8, decreased to 2.4 ± 1.8 (*p* < 0.001) at the 3 to 6 month visit and was as well improved to 3.3 ± 2.7 (*p* < 0.001) at last follow-up. A worsening of neck pain during follow up was observed only in one patient (2.2 %).

#### Arm pain

The mean VAS score for arm pain improved significantly from 5.9 ± 3.1 preoperatively to 1.9 ± 1.8 (*p* < 0.001) at 3 to 6 months postoperatively and to 2.8 ± 2.7 at the last follow-up (*p* < 0.001) (Table 1).

#### Used NSAIDs

Concerning the use of NSAIDs, preoperatively 51.9 % of the patients “always”, 14.8 % “often”, 14.8 % “seldom” and 18.5 % “never” used painkillers (Table 1). At the 3 to 6 months follow up 48.9 % of the patients “never”, 23.3 % “seldom”, 16.3 % “often” and 9.3 % “always” used NSAIDs (*p* < 0.001). At the last follow-up a significant reduction was observed as well. 45.8 % of the patients rated their use of painkillers with “never”, 33.3 % with “seldom”, 8.3 % with “often” and 12.5 % with “always” (*p* < 0.001).

#### Overall success

The analyses of patient overall satisfaction (Table 2) at last follow-up showed good to excellent results in 95.7 % of the cases. In detail: 34.8 % showed excellent improvement. 43.5 % of the patients quoted the postoperative result as significantly better, 19.5 % rated their physical condition as good. 2.2 % (one patient) described no improvement of his ailments. No patient showed a worsening at last follow up. Concerning PSI at 3 to 6 months follow-up 65.9 % of the patients answered the question, “Would you like to have the same treatment for the same ailment again?” with “definitely yes” and 31.7 % of the patients with “probably yes”. One patient (2.2 %) answered the question with “probably not”.

**Table 2:** Overall Satisfaction and PSI

	Before discharge	3 to 6 month	Last follow-up
Overall Satisfaction			
Excellent Improvement	9.5 %	23.8 %	34.8 %
Significantly better	71.4 %	64.3 %	43.5 %
Good Condition	19.0 %	11.9 %	19.5 %
No Improvement	--	--	--
Worsening	--	--	2.2 %
PSI			
“Would you have the same treatment for the same ailment again?”	80 % “definitely yes” 20 % “probably yes”	65.9 % “definitely yes” 31.9 % “probably yes” 2.2 % “probably not”	77.3 % “definitely yes” 22.7 % “probably yes”

NOTE. All values are presented as percentage of patients

### Radiographic evaluation

**Fig. 2:**
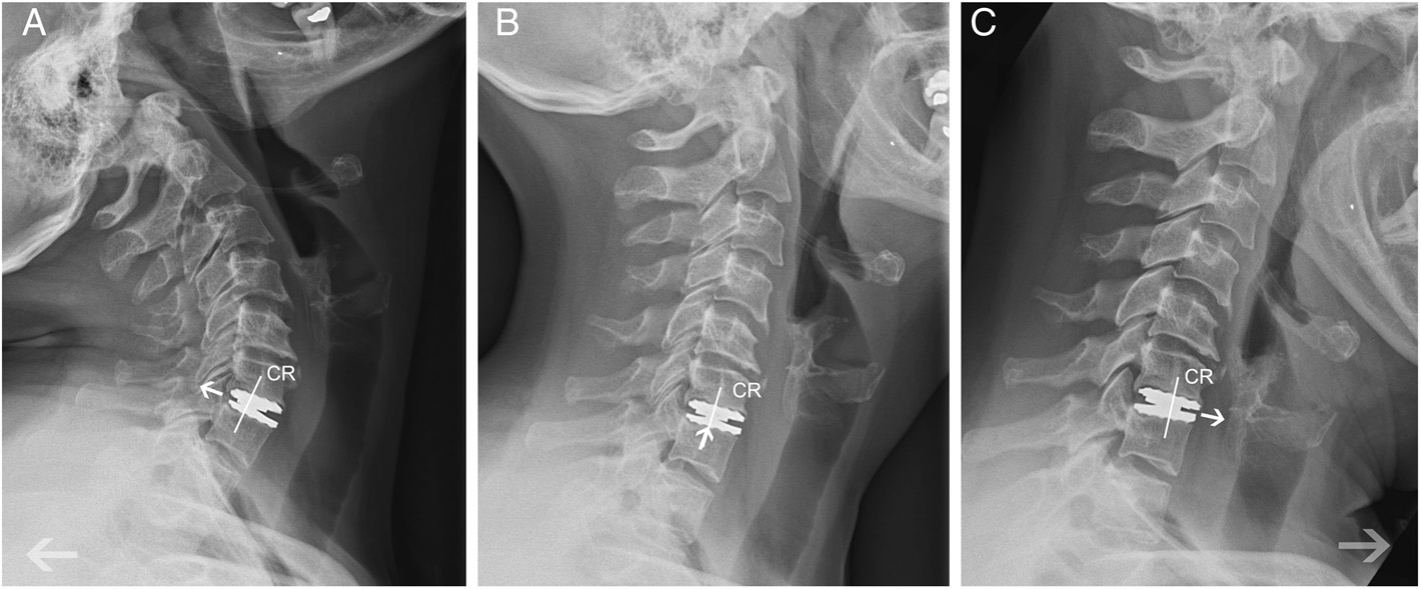
Flexion (**a**), neutral (**b**) and extension (**c**) x-ray showing the successful operation – especially the variable mechanical centre of rotation (CR) of the device moving forward and backward (white arrows) - of a ROTAIO cervical disc prosthesis at the level C6-C7 in a 52 years old female patient, 6 month after implantation

**Fig. 3:**
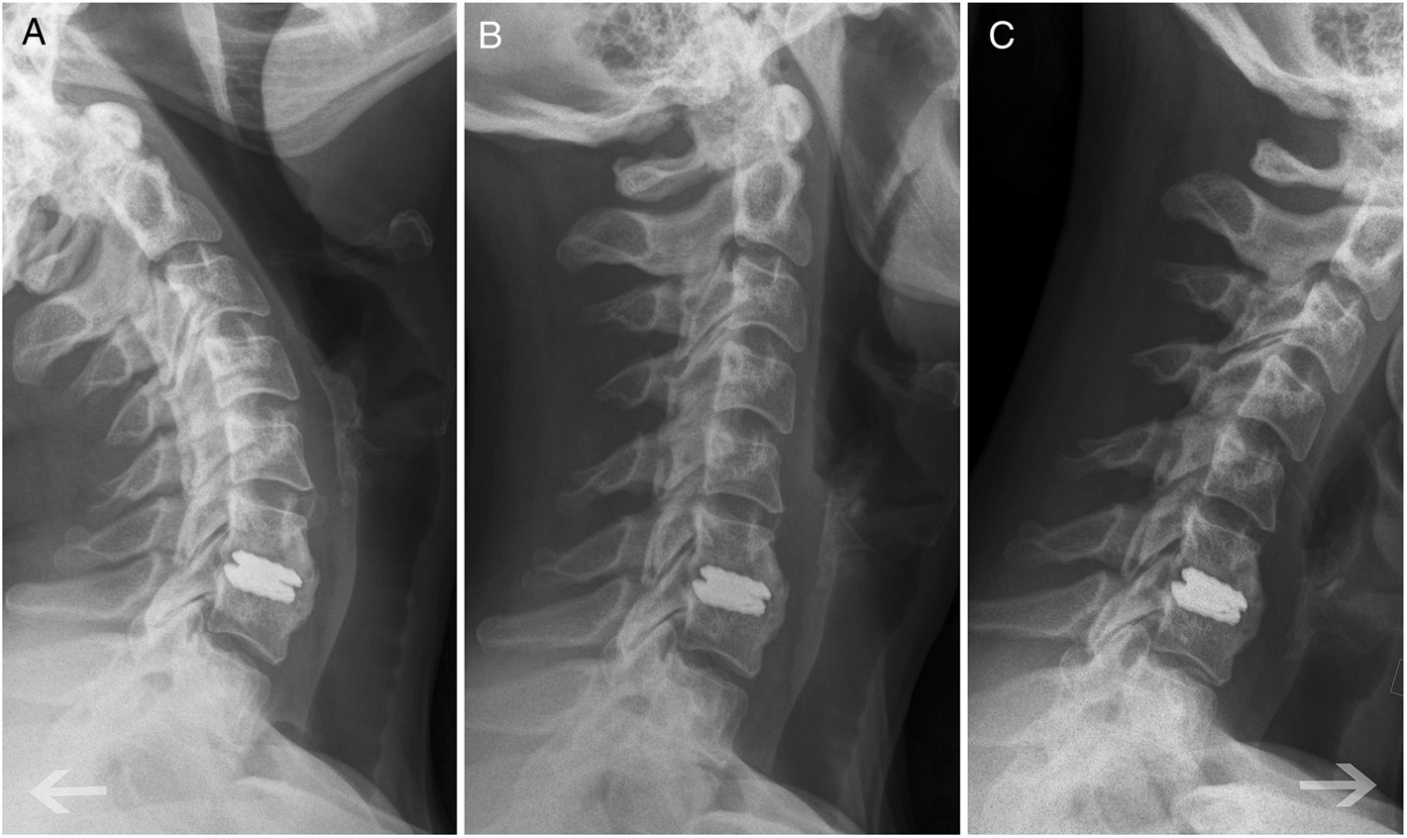
Flexion (**a**), neutral (**b**) and extension (**c**) x-ray showing an immobilized ROTAIO cervical disc prosthesis due to heterotopic ossification at the level C6-C7 in a 48 years old female patient, 12 month after implantation

Forty-one patients could be enrolled to radiological follow-up measurements (Fig. 2). There were no signs of anterior migration or dislocation of the prosthesis. Subsidence was defined as a decrease of more than 2 mm in functional spinal unit (FSU) from 3 months to last follow-up and was observed in no patient. The sagittal alignment was well restored in all cases. In one case (2.2 %) the radiographical one-year follow-up showed a bridging bone ventrally to the prosthesis and mild signs of adjacent segment disease like new anterior osteophytes (Fig. 3). Overall the analyses of dynamic flexion and extension X-rays showed no malfunction of the prosthesis. Concerning the ROM a significantly increased motion was detected at every time point. The mean cervical spine motion in flexion-extension at the index level improved from 6.3° ± 2.9° preoperatively to 8.4° ± 2.5° at 3 to 6 month after surgery (*p* < 0.001) and to 8.6° ± 2.8° at last follow-up (*p* < 0.001).

### Complications

Self-limiting dysphagia was detected in three patients, which was probably due to traction or laryngeal nerve irritation during surgery. There was no other minor or major complication and no implant-related complication such as subsidence or fractured vertebrae.

#### Secondary procedures

**Fig. 4:**
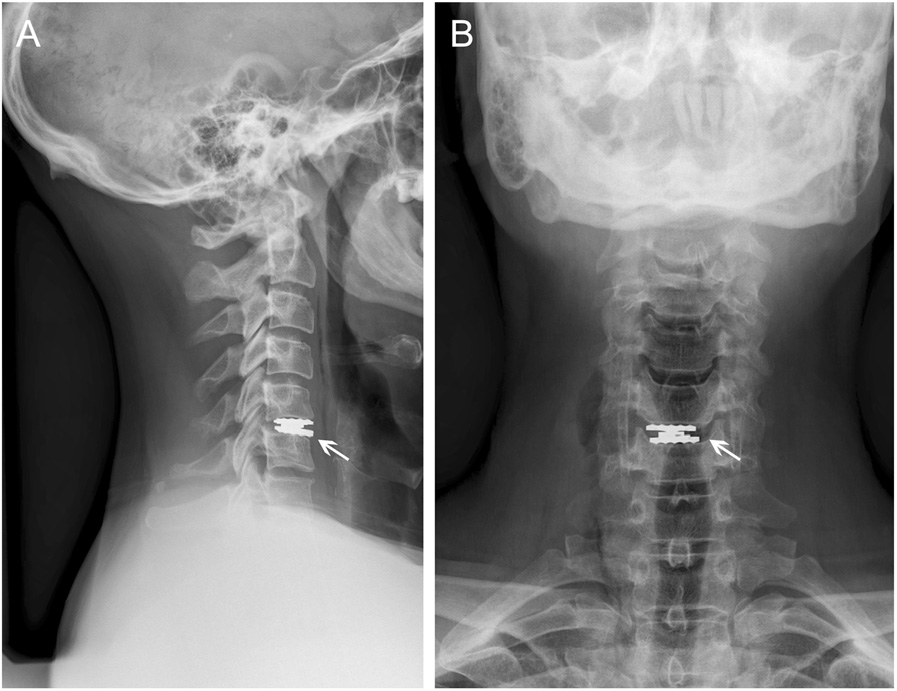
X-ray in neutral position (**a**: lateral view; **b**: anterior view) showing an undersized and too ventrally positioned ROTAIO cervical disc prosthesis at the level C5-C6 (white arrows) in a 38 years old male patient, 3 month after implantation. Within the first 12 month of follow up the patient suffered from progressive neck pain

In one male patient revision surgery at the index level was required (2.2 %). Caused by implantation of an undersized and too ventrally positioned prosthesis the patient suffered from increasing neck pain (Fig. 4). A few weeks after the 12 months follow-up the prosthesis was removed and the patient was treated with ACDF.

## Discussion

ACDF is a successful treatment and has shown high patient satisfaction rates for years. During the last two decades the effectiveness of CDR in treating cervical degenerative disc diseases has been analysed and in summary CDR after anterior neurological decompression may be deemed as an alternative to fusion in selected patients [5, 8–11]. Till now biomechanics, clinical and radiological effectiveness of most current available artificial discs prosthesis have been studied [1, 4, 5, 12–14]. As a newly established device the ROTAIO cervical disc prosthesis has only been in use for a few years. This is the first prospective study that analyses its clinical and radiographic reliability.

The results of this study demonstrated promising clinical and radiological outcomes. Concerning the NDI the observation showed a significant improvement of 51.5 % (*p* < 0.001) after implantation which correlates with present studies of other available cervical disc prosthesis [4–6]. Analysis of VAS showed a significant reduction of neck and arm pain early after surgery which might primarily be related to sufficient decompression during surgery and the in-hospital pain management. At last follow-up the improvement of arm and neck pain was significant as well and was also confirmed by a significant reduction of used NSAIDs. Regarding overall satisfaction 95.7 % of the patients achieved good to excellent improvement which is in-line with reports that observed the early results of other established cervical disc prostheses and also ACDFs [1, 3, 5, 8, 9, 11, 15–18]. High patient satisfaction rates were also reinforced by evaluating the PSI which has shown that 77.3 % of the patients would definitely have liked to have the same treatment for the same ailment again at the last follow up.

According to prior clinical trials of cervical arthroplasty subsequent secondary surgical intervention was defined as any revision, removal, reoperation or supplemental fixation [1, 4–6]. Until the last follow-up in one case (2.2 %) an increase of neck and arm pain was observed and subsequent secondary surgical intervention was required to remove a probably undersized prosthesis. As published by Sasso et al [1], Burkus et al [7] and Anderson and Hashimoto [19] for other disc prostheses the results of this study show a very low rate of secondary procedures and no prostheses related complications during or after surgery. According to Li et al [6] proper selection of patients, exact intraoperative strategy and adequate neural decompression is required to achieve satisfactory clinical outcomes.

Radiographic analysis showed no dislocation, anterior migration or malfunction of the prosthesis. As reported by Heller et al ROM values measured radiographically can be influenced by the radiographic technique itself, patient´s motivation or other – unknown – factors [5]. Nevertheless most authors observed motion preservation at the implanted level and characterized it as one of the fundamental benefits of CDR [1, 3, 4, 16, 20]. In the present study the prosthesis maintained physiological segmental motion with a mean flexion-extension difference of 8.4° in the 3 to 6 month visit and 8.6° at the last follow-up. According to Heller et al [4] an increasing ROM probably is a sign of less pain and higher patient´s motivation. Additionally a restored lordotic sagittal alignment was observed in all cases thus compared to other available CDR devices the ROTAIO prosthesis does not show a lordotic design like e.g. the Discover disc [6]. A fact that might be an advantage over prostheses like the Bryan disc, for which segmental kyphosis of the index level – persisting for more than 6 months postoperatively - was reported by Pickett et al [20]. The radiographic analysis also showed a low rate (2.2 %) of spontaneous fusion as measured by presence of bridging bone. This result may be a consequence of the preserved ROM but according to the works of Heller et al [5] it could also be influenced by patient selection. The authors describe that patients with significant spondylosis may have a greater likelihood of spontaneous fusion [5].

Adjacent segment disease (ASD) following uninstrumented ACDF has been reported to occur in 25 % of patients within 10 years and shows an incidence of 2.9 % per year [21, 22]. Based on the rationale that CDR helps to preserve the biomechanics of the spine in the index level and in addition also in the adjacent segments the potential for decreasing ASD was one of the most striking arguments for total disc arthroplasty. As reported by Riew et al [22] it is quite challenging to compare rates of radiographic or symptomatic ASD across the available literature because authors use substantially different definitions for their analysis and because there is no direct relationship between radiographic and symptomatic ASD. In the present study radiographic ASD was defined as new anterior osteophyte formation or enlargement of existing osteophytes and the rate of reoperation at the level directly adjacent to the treated level. In one case (2.2 %) anterior bridging bone at the index level and new osteophyte formations in the adjacent cranial level was observed. The low rate of ASD goes in line with the results of other authors and may be artificially low because of the definition as mentioned above and the follow up with a maximum period of just 24 month [1, 7, 22, 23] thus e.g. Park et al [24] reported that adjacent-level anterior disc space ossification (ALOD) occurs within the first 12 months after treatment and that patients with no ossification at 24 months are very unlikely to progress to advanced ossification. Furthermore - according to the work of Nunley et al [25] – over and above the device design there may be lots of different factors influencing the development of an ASD like e.g. age, sex, smoking habit or coexisting diseases.

Concerning the development of symptomatic ASD the results of this study show a mild increase of the mean VAS regarding neck pain from the 6 months visit to the last follow up ( 2.42 to 3.33; *p* > 0.05). Nevertheless this may be associated to the prosthesis or may be a part of degenerative spine´s natural history, long-term follow-up is necessary to evaluate if differences in adjacent segment motion and radiographical degeneration will affect the development of clinical ASD. To what extent the new design of the ROTAIO cervical disc prosthesis is able to reduce the risk of ASD and how far the device could protect the uncovertebral and facet joints from overload must be the aim of upcoming biomechanical analysis.

Overall new technology such as artificial disc devices requires long-term follow up to assess durability, the biologic compatibility and the response of the prosthesis to its environment. Typically failure of other joint arthroplasty does not occur before five to ten years after surgery. Therefore spinal disc prostheses similarly need to be assessed repetitively and for a longer period [1, 18]. Nevertheless based on clinical outcome according to the significant reduction of NDI and VAS scores, the high overall satisfaction and the evaluated safety of the device the ROTAIO prosthesis may be deemed as a good alternative for currently available cervical disc prostheses.

### Limitations

There are some limitations in this study including the small number of patients, the lack of an ACDF control group and the limited follow up. Moreover the biomechanical behaviour of the ROTAIO disc in axial rotation and lateral bending has not been evaluated. Additionally the comparison with other kinds of artificial discs may provide further insights into the characteristics of different CDR devices.

## Conclusions

The early results of this prospective, observational study show that cervical disc arthroplasty with the ROTAIO Cervical Disc Prosthesis seems to be safe and effective for the treatment of symptomatic single-level degenerative disc disease. Additionally patients had significantly improved NDI and VAS scores, showed a lower rate of NSAID-use and an excellent overall success at last follow-up. Thus, according to recent studies of other cervical disc prostheses the results confirm and support the role of total disc replacement as an alternative to ACDF, studies focusing on clinical long-term outcome and especially on preserving ASD are warranted.

## Abbreviations

CDA: Cervical disc arthroplasty
ACDF: Anterior cervical decompression and fusion
ASD: Adjacent segment disease
NDI: Neck disability index
VAS: Visual analogue scale
ROM: Range of motion
FSU: Functional spinal unit
C1-n: Cervical vertebra 1-n
NSAID: Non-steroidal anti-inflammatory drugs
CDR: Cervical disc replacement
PSI: Patient satisfaction index
